# Design of Amphibious Vehicle for Unmanned Mission in Water Quality Monitoring Using Internet of Things

**DOI:** 10.3390/s18103318

**Published:** 2018-10-03

**Authors:** Balasubramanian Esakki, Surendar Ganesan, Silambarasan Mathiyazhagan, Kanagachidambaresan Ramasubramanian, Bhuvaneshwaran Gnanasekaran, Byungrak Son, Su Woo Park, Jae Sung Choi

**Affiliations:** 1 Centre for Autonomous System Research, VelTech Rangarajan Dr. Sagunthala R&D Institute of Science and Technology, Avadi, Chennai 600 062, India; balasubramaniane@veltech.edu.in (B.E.); surendarg@veltechuniv.edu.in (S.G.); silambu.friends@gmail.com (S.M.); 2 Department of Computer Science Engineering, VelTech Rangarajan Dr Sagunthala R&D Institute of Science and Technology, Avadi, Chennai 600 062, India; drgrkanagachidambaresan@veltech.edu.in; 3 UCAL System Ltd., Chennai 600 018, India; gd.bhuvanesh1@gmail.com; 4 Convergence Research Center for Wellness, DGIST, Daegu 42988, Korea; 5 Rovitek Inc., 91, Damun-ro 61-gil, Jillyang-eup, Gyeongsan-si, Gyeongsangbuk-do 38479, Korea; swpark@rovitek.com; 6 Department of Computer Engineering, Sun Moon University, Asan-si 31460, Korea; jschoi@sunmoon.ac.kr

**Keywords:** amphibious UAV, hovercraft, FEA, CFD, prototype, water quality, sensors, Internet of Things

## Abstract

Unmanned aerial vehicles (UAVs) have gained significant attention in recent times due to their suitability for a wide variety of civil, military, and societal missions. Development of an unmanned amphibious vehicle integrating the features of a multi-rotor UAV and a hovercraft is the focus of the present study. Components and subsystems of the amphibious vehicle are developed with due consideration for aerodynamic, structural, and environmental aspects. Finite element analysis (FEA) on static thrust conditions and skirt pressure are performed to evaluate the strength of the structure. For diverse wind conditions and angles of attack (AOA), computational fluid dynamic (CFD) analysis is carried out to assess the effect of drag and suitable design modification is suggested. A prototype is built with a 7 kg payload capacity and successfully tested for stable operations in flight and water-borne modes. Internet of things (IoT) based water quality measurement is performed in a typical lake and water quality is measured using pH, dissolved oxygen (DO), turbidity, and electrical conductivity (EC) sensors. The developed vehicle is expected to meet functional requirements of disaster missions catering to the water quality monitoring of large water bodies.

## 1. Introduction

Unmanned aerial vehicles (UAVs) are categorized based on the performance characteristics of their wing movement such as fixed-wing, rotary, and flapping-wing configurations [1]. Various applications of UAVs include surveillance, traffic monitoring, active weapon engagement, wildlife surveying, pollution monitoring, precision agriculture, etc. [2]. Authors of this paper contributed to the development of UAVs for environmental monitoring [3], structural health monitoring [4], and also constructed micro aerial vehicles [5,6,7]. Dedicated efforts on the development of amphibious vehicles are scarce. Collins [8] described the importance of amphibious UAV in diverse applications and discussed their relevance in issues pertaining to control, communication, and airspace management. Boxerbaum et al. [9] developed a robotic amphibious vehicle using biological concepts inspired by animals to navigate in underwater and rough terrains. Yayla et al. [10] performed theoretical analysis to investigate the performance characteristics such as rate-of-climb, turn radius, and maximum velocity of an amphibious UAV. Pisanich and Morris [11] fabricated a sea plane conceptual model of an amphibious UAV for a 4 kg payload. Autonomous flight missions were performed in air and water as a proof of concept. Hasnan and Wahab [12] designed a UAV that can fly in air, and glide along land and a water surface. Frejek and Nokleby [13] designed a four-paddle-wheel amphibious vehicle with ultrasonic sensors to detect obstacles. The published data indicates that development of amphibious UAVs for deployment in water quality assessment is not evident from the literature. Measurement of water quality is usually performed with the aid of boats [14], which is labor-intensive and costly. Remote sensing methods of water quality assessment is time-consuming and needs lot of investment [15]. Traditionally, water body agencies are collecting the water samples manually and in a periodic manner, which is cumbersome [16]. Few of the lakes, rivers, ponds, and reservoirs may not have access to collect water samples with boats and they might be surrounded with shrubs and bushes [17]. Also, in situ measurement of water quality at the designated locations of water bodies and periodic measurement are of paramount interest to address the water quality [18]. An autonomous underwater vehicle is deployed [19] and it suffers due to the global positioning system (GPS) denied environment and controlling of those vehicles are difficult to collect water samples at the precise water location. In order to overcome the aforementioned difficulties, a radical approach in collecting water samples and performing in situ water quality assessment is necessary. UAV-based water quality can be a relatively inexpensive, easy, and effective approach in accessing the large water bodies in a short span of time. Landing of vehicles in dangerous water locations where human threat is of major concern and inaccessible water bodies with boats are effortlessly accessed with the aid of UAVs. Also, moving along the water body may be of peculiar interest to consume less power that necessitate UAV with amphibious characteristics. For quality evaluation of large and inaccessible water bodies, amphibious vehicles provide effective and rapid solutions. Development of a UAV that can land and glide on the water’s surface while collecting water samples offers several challenges related to materials, energy management, control systems, and on-board sensors. The present study integrates features of a multi-rotor UAV with a hovercraft and this configuration for an amphibious UAV has not been attempted yet. The vertical take-off and landing (VTOL) functionality is also integrated into the system so that the resultant amphibious vehicle offers several functional advantages, such as energy efficient movement on the water’s surface, eliminating large areas for landing and take-off besides ensuring compatibility with a wide variety of payloads.

## 2. Evolving Conceptual Model

**Figure 1:**
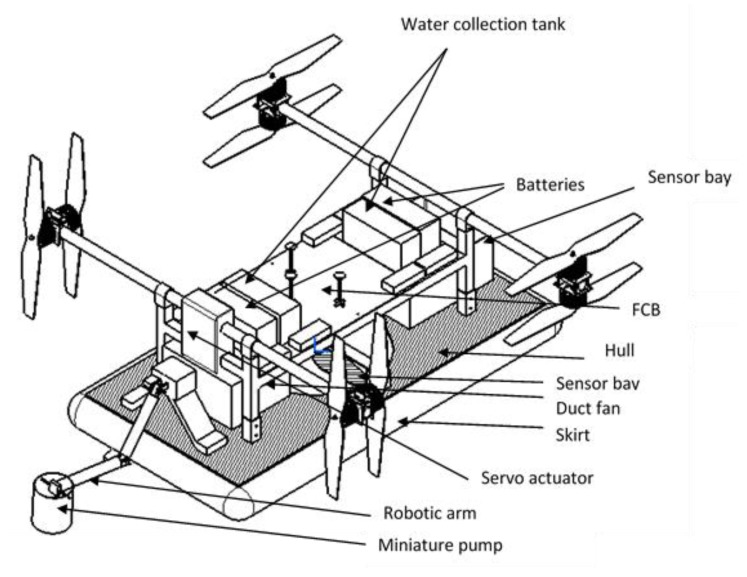
Conceptual model of amphibious vehicle.

The conceptual model is formulated by integrating the multirotor and hovercraft configurations wherein four co-axial propellers and motors that are attached to the frame act as an octo-rotor as shown in Figure 1. The entire rotor assembly is supported by a hull made of high-density polyurethane foam, and nylon impregnated with urethane is attached beneath for functioning as a skirt. Provision for payloads, batteries, sensors, electronic accessories, flight controller board (FCB), water sampler with robotic arm module, and water collection tanks are made in such a way that the center of gravity (CG) of the vehicle is maintained for stable flight.

The co-axial propellers are actuated during vertical take-off and landing. After landing on a water surface, any two co-axial propellers are rotated through 90° using a servo motor and these propellers produce thrust for forward movement of the vehicle. Buoyancy of the vehicle is achieved through a cushioning effect of the skirt produced using a duct fan. A hover gap of 2–5 mm is maintained between the skirt of amphibious vehicle and the water surface. During hovering mode, all four co-axial motors are powered to create lift. 

In addition to these design requirements, the following performance specifications are considered for selection of aircraft components:

- Airborne operation
  - Flight endurance of 20 min
  - Payload of 7 kg
  - 40 km/h cruise speed
  - 2000 m of flight range
- Hovering and moving on water body
  - Endurance of 40 min
  - 30 km/h cruise speed
  - 2000 m range

Based on these design requirements and performance criteria, a mission profile for the collection of water samples in remote water body locations is identified. The typical mission profile has flight conditions of VTOL, hovering on air, landing on water bodies, propelling along the water surface, and vertical landing. The sequence of these missions is varied according to the operational need.

## 3. Design Process

**Figure 2:**
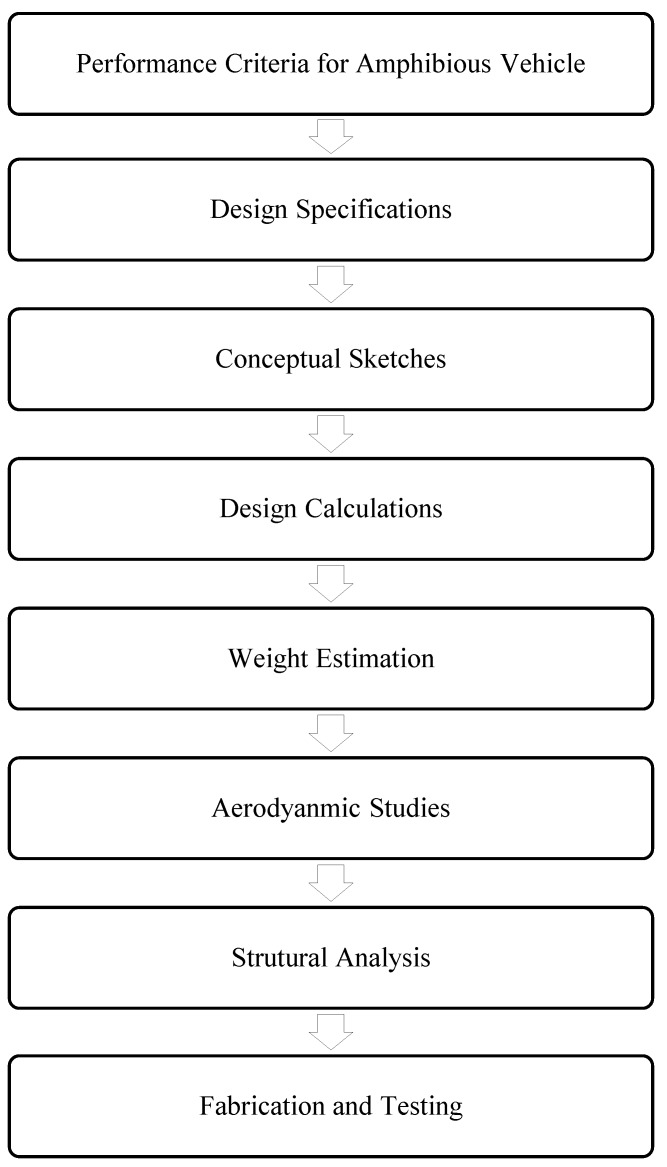
Design strategy of the amphibious vehicle.

Design process of the amphibious vehicle capturing the functionalities corresponding to aerodynamics, structures, and compliance to performance criteria is presented in Figure 2.

### 3.1. Design of Hovercraft

A hovercraft is an air cushion vehicle that moves over multiple terrains including land, water, and muddy surfaces. The duct fan located at the center produces the necessary cushioning effect through forcing air down and creating an air cushion between the skirt and the water surface. Inflation of the skirt increases air pressure that acts at the base of the hull. Forward motion of the hovercraft is achieved through propelling the co-axial rotors. Since the skirt is considered to be a sensitive part of the hovercraft to achieve lift of the vehicle, selection of skirt material is an important aspect that is discussed in the following section. In order to design a hovercraft, various parameters have to be determined. Table 1 presents the list of assumptions incorporated to perform design calculations, and in Table 2 hovercraft parameters are estimated for the flow analysis [20].

#### 3.1.1. Selection of Skirt Material

The inflation of skirt functionality for realization of hovering means the skirt material should have sufficient tensile strength. A survey of various skirt materials [21,22,23,24,25] for hovercrafts given in Table 3 reveals that nylon impregnated with urethane was the best choice due to its high tensile strength, light weight, and superior resistance to wear and tear characteristics.

#### 3.1.2. Selection of Hull Material

The hull is considered to be a watertight body part of the hovercraft and it has to support various payloads, the battery, and other electronic systems. It has to withstand high upward pressure generated through the cushion of air during the inflation of the skirt. Based on the survey of material [21,22,23,24,25] options (Table 4), polyurethane foam was selected due to its high strength-to-weight ratio.

### 3.2. Multicopter Design

A hollow square cross-section aluminium channel was considered for the horizontal and vertical frames for supporting the pair of motors. The speed and thrust of the motors [26] were calculated (Table 5) using empirical relations.

#### 3.2.1. Propulsion

As per the initial estimation of the speed of the motor, the motor was selected with reference to the Kv rating (125 Kv) having a power of 1900 W. Table 6 illustrates the necessary current rating and number of cells of a battery for 25 min of endurance for a battery capacity of 22,000 mAh.

#### 3.2.2. Selection of Propeller

**Figure 3:**
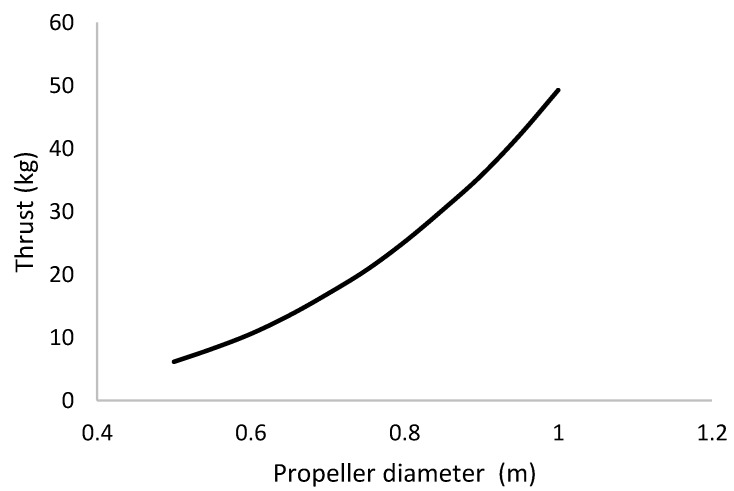
Selection of the co-axial propeller diameter.

The weight of amphibious system was considered to be 30 kg for selection of the propellers. A Quad with a co-axial motor–propeller configuration was considered due to the demand of high payload-carrying capacity and stability of the vehicle. Considering the thrust to weight ratio as 2% and a 20% thrust loss due to the co-axial configuration, the maximum thrust was estimated as 75 kg. Under the full-throttle condition, propellers of various diameters and their thrust force [27] characteristics were examined (Figure 3). In order to lift 75 kg, each co-axial arm needed to produce approximately 18.5 kg of thrust. Hence, a co-axial propeller configuration with diameter 0.75–0.80 m was selected.

#### 3.2.3. Selection of the Motor

**Figure 4:**
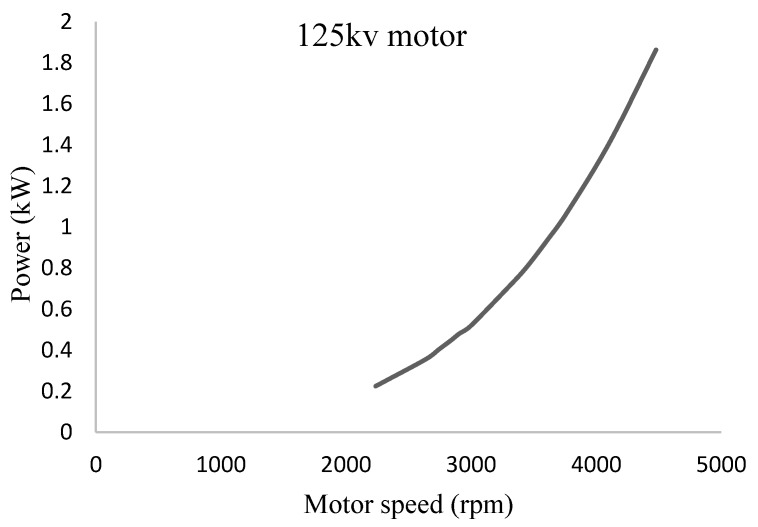
Power consumption characteristics of the selected motor.

Selection of the motor primarily depends upon the size of the propeller for generating sufficient thrust, and the selected configuration demands 10 kg thrust force per motor. Power consumption of the 125 kv motor under various speeds was determined as shown in Figure 4. Under a full-throttle condition, a maximum power of 1.86 kW is required per each motor at a speed of 4480 rpm. 

In this work, a T-Motor MN705-S (T-Motor, Nanchang, China) with 125 Kv, ideal current of 1.4 A, peak current of 45 A, and maximum power of 2200 W was considered [28]. 

#### 3.2.4. Selection of the Battery

Selection of a battery depends upon the consumption of current with sufficient voltage and discharge rate requirements. Total current consumption for the electronic components was calculated to be 2.73 A (Table 7). Considering eight motors, the total power and current required for the vehicle to fly in the air were estimated as 3.78 kW and 79.13 A (Table 8), respectively. However, when the vehicle lands on water and glides along the water surface, two pairs of motors need to be actuated. Estimation of current and power consumption during the gliding of vehicle on the water surface is given in Table 9 and it is evident that only half of the power is required for the amphibious mode as compared to the flight mode.

In order to meet these power and current consumption requirements, a 22,000 mAh capacity battery was selected. It was expected to have an estimated endurance of 22 min while in air flight and 46 min during hovering or gliding along the water body.

### 3.3. Weight Estimation

Based upon earlier selection of materials for hovercraft and multirotor components, weight of the amphibious vehicle is estimated as 27.31 kg inclusive of 7 kg payload. Table 10 shows the weight of various components of hovercraft and multirotor systems.

## 4. Structural Analysis of the Amphibious Vehicle

**Figure 5:**
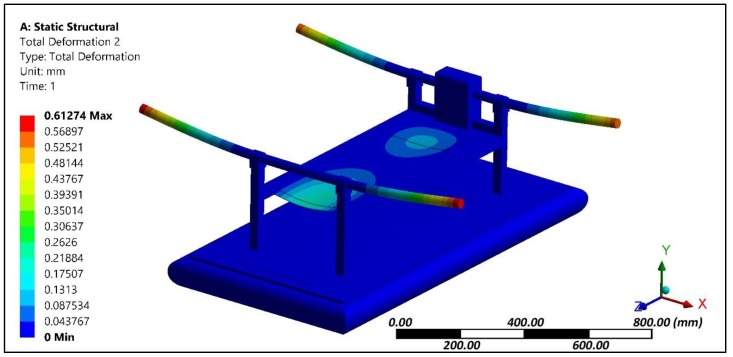
Deformation plot of the amphibious structure.

**Figure 6:**
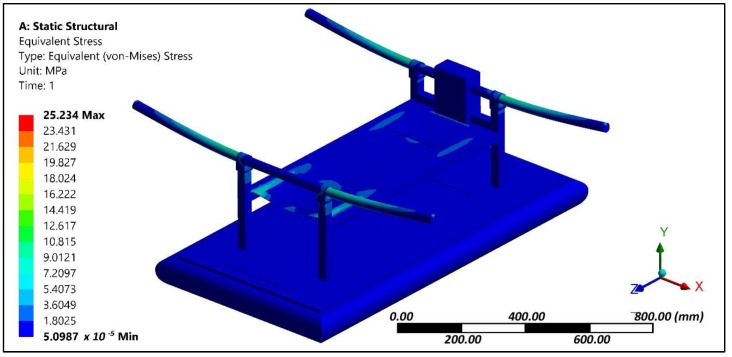
Stress contour of the amphibious structure.

The multirotor configuration had vertical and horizontal frames that were made of aluminium channels owing to its lightweight characteristics. At the tip of the horizontal frames, the motor and co-axial propellers were attached. The vertical frames were anchored to the top surface of the hovercraft hull. The thrust produced by the propellers [29,30,31] was considered to be acting at the fixed support of horizontal frame and the same vertical axial loading was applied at the four corners of the horizontal frame. An axial load was applied at the tip of the frame and the effect of cushion pressure generated through the duct fan located at the center of hull was analyzed. The pressure load was applied at the inner surface of the skirt and bottom of the hull. The effect of these loading conditions was evaluated through structural analysis. A displacement of 0.6 mm was experienced at the tip of horizontal frame (Figure 5). The von Mises stress plot (Figure 6) shows that the junction of horizontal and vertical frames experiences maximum stress regions about 25 MPa. Other portions of the amphibious structure experienced considerably lower levels of stress.

## 5. Aerodynamic Analysis

**Figure 7:**
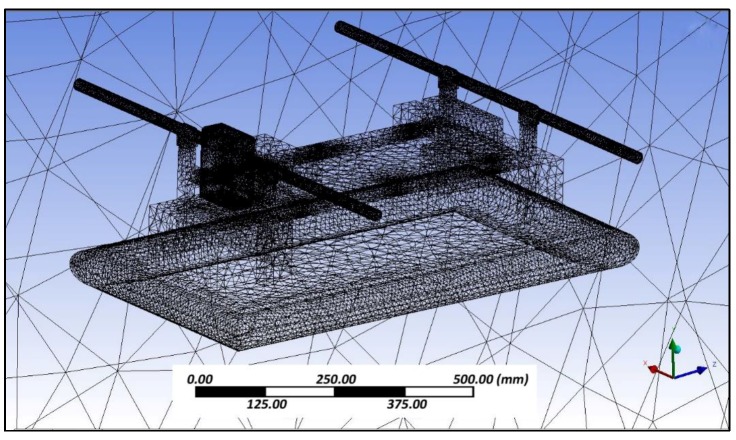
Meshed geometry.

Aerodynamic evaluation of the amphibious vehicle was performed through varying the wind speeds in the range of 5 to 10 m/s with different angles of attack (AOA) (0° and 8°). Computational fluid dynamic (CFD) analysis using the ANSYS FLUENT platform (ANSYS, Canonsburg, PA, USA) was used to examine the velocity and pressure contours during forward flight conditions, and aerodynamic coefficients were also determined. The quality of meshing (Figure 7) is evaluated through performing orthogonality and skewness characteristics (0.9). Inlet as velocity and outlet as a pressure was considered and boundaries were defined far away (10 times) to reduce the horizontal buoyancy effect and wall inference. A symmetric plane and no heat transfer were assumed to perform simulations.

**Figure 8:**
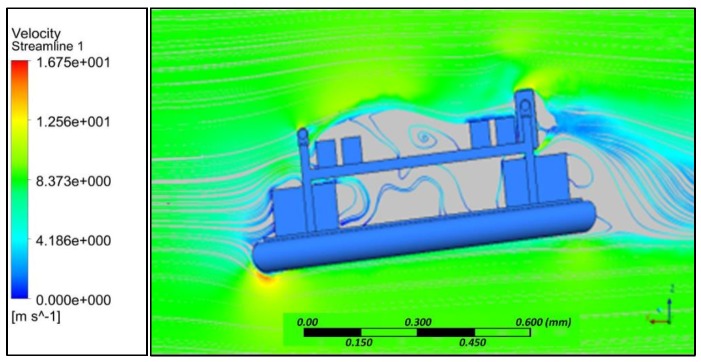
Velocity streamline at 4° AoA.

**Figure 9:**
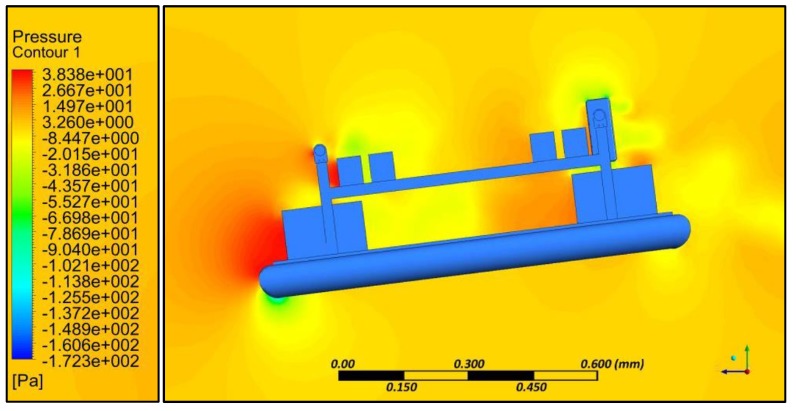
Pressure contour at 4° AoA.

Simulation results indicated that at various angles of attack, collision of air with the frontal body surface caused a velocity drop (Figure 8) due to stagnation pressure (Figure 9), and there was a loss of kinetic energy. At the middle of the amphibious vehicle, a low-pressure region formed that created vortex and flow separation. This phenomenon may have created an imbalance of the vehicle, which can be streamlined through providing riblets. At the rear of the vehicle, recirculation flow occurred due to non-uniformity and a blunt profile of the UAV structure.

**Figure 10:**
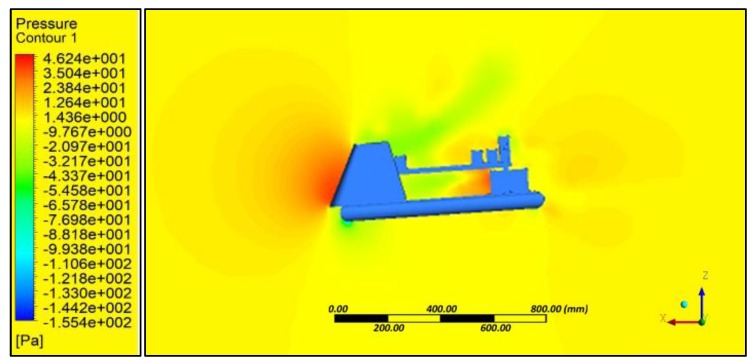
Flat panel—Pressure contour.

**Figure 11:**
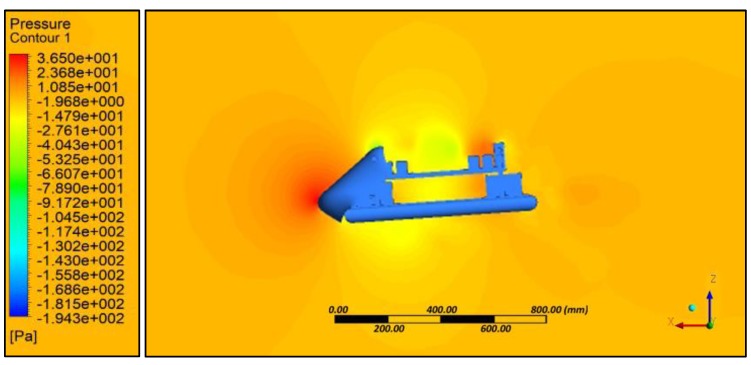
Blended nose—Pressure contour.

For various angles of attack, the co-efficient of drag was estimated and the corresponding drag force was calculated (Table 11). It is evident that substantial drag was experienced, which reduced the endurance of the UAV. In order to reduce the effect of drag, an inclined front panel (Figure 10) and blended nose configurations (Figure 11) were considered.

## 6. Water Sample Collection Using Robotic Arm

**Figure 12:**
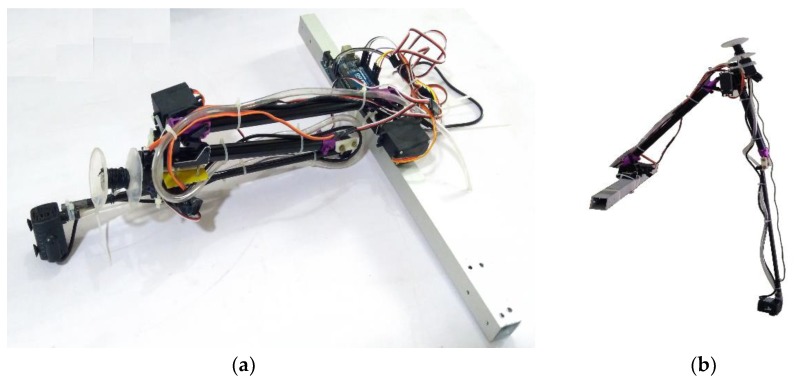
Robotic arm with suction pump assembly, (**a**) Folded arm (**b**) Extended arm.

A two-degree-of-freedom (DOF) manipulator actuated using a servo motor was used to collect water samples as shown in Figure 12. An end-effector carried a water-sucking pump, which in turn connected through a hose. Drawn water was collected in the respective storage tank with a 1 L capacity. The depth of water collection was controlled using a rope driven by a stepper motor. Encoder feedback was sent to an Arduino based controller (Arduino Srl, Strambino, Italy) to monitor the depth of the collection of water. The arm of robot manipulator was made up of carbon fiber and a waterproof servo motor was attached at each link of the robotic arm. During the water sample collection, stability of the vehicle was assured through distributing water using a two-way control value. A water level sensor was used to measure the quantity of water, and corresponding feedback was sent to control the pump and retraction of the robotic arm. Buffer plates were placed in the water storage tank to dampen the vibration caused due to turbulence of water in the storage tank.

**Figure 13:**
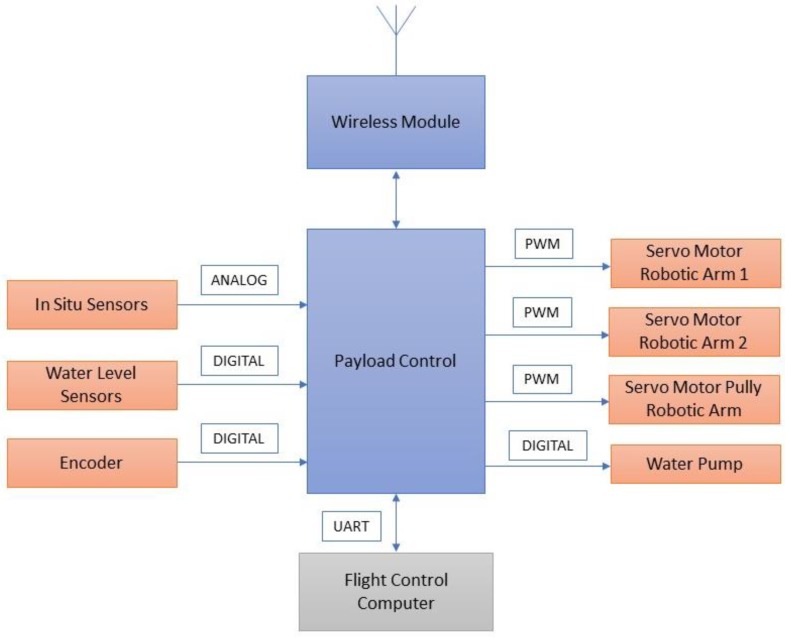
Main control unit.

Figure 13 illustrates the payload control unit, in which pulse-width modulated (PWM) signals were sent to actuate the servo motors. The water level sensors, water quality monitoring sensors, pump, and encoder were used to provide feedback in analog and digital forms.

## 7. Development of Ground Control Station

**Figure 14:**
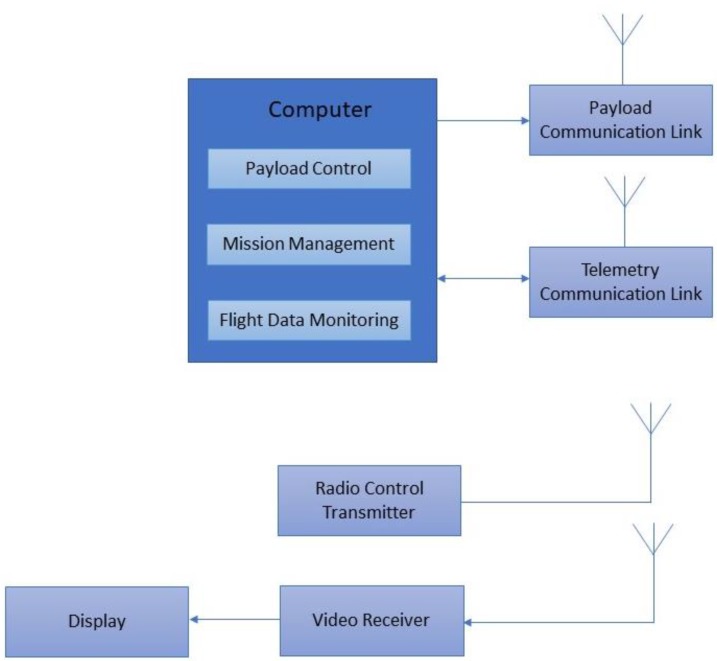
Ground control station.

The ground control station (Figure 14) consisted of a portable computer with payload control, mission management, and flight data monitoring with corresponding communication links. The mission of the amphibious vehicle was pre-planned in the ground control station wherein the vehicle was flown as multi-rotor to identify contaminated regions of the waterbody and on-line video was streamed using a 5.8 GHz video data link. Once contaminated regions are identified, the vehicle was landed on the water surface through the hovercraft mode. Water samples were collected using a robotic arm with suction pump and rope mechanism structure. Radio frequency signals in universal asynchronous receiver—transmitter (UART) carrier mode were used to communicate and actuate the servos, sensors, and other actuators to collect required water samples with precise feedback.

**Figure 15:**
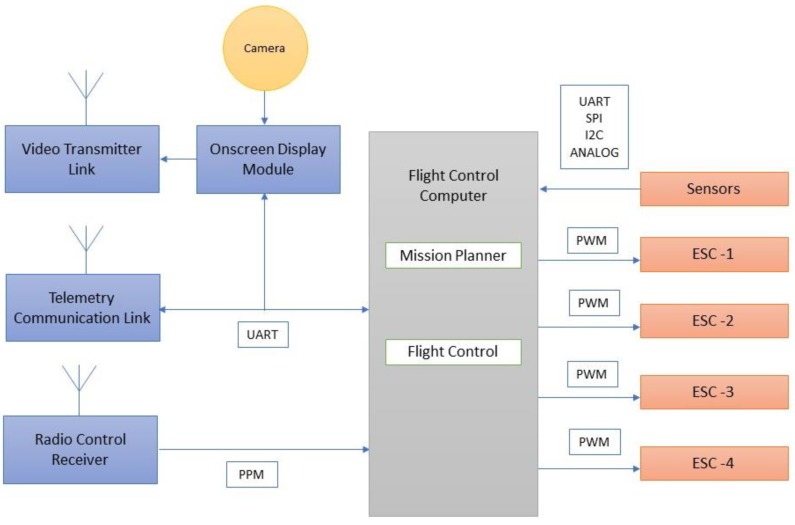
Flight control computer module.

A typical flight control computer [32] is presented in Figure 15. It acted as the central hub of the system through which position, orientation, and heading direction of the vehicle were controlled. In addition, the receiving and transmission of data, battery power monitoring, and actuation of servos, motors, and the robotic arm were performed.

**Figure 16:**
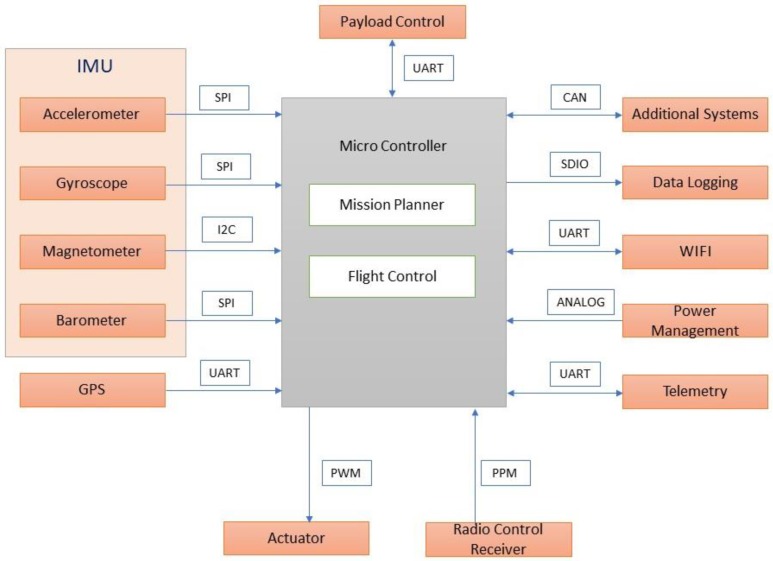
Airborne mission.

The airborne mode of the mission is depicted in Figure 16 and explains that the radio frequency signals at 2.4 GHz were transmitted and received through telemetry modules. The received signals are sent in pulse position modulated (PPM) form to the flight controller. The flight controller computer handled control and navigation of the vehicle during flying and hovering modes. PWM signal from flight control computer is sent to the electronic speed controller (ESC) to actuate the brushless direct current (BLDC) motor to lift and navigate the vehicle in the desired attitude and altitude. The on-line video streaming was achieved through RF mode and on-screen display module is integrated to monitor the water surface in real time during flying mode. Autonomous capability was achieved through way point navigation, guidance, and control with prior mission planning.

**Figure 17:**
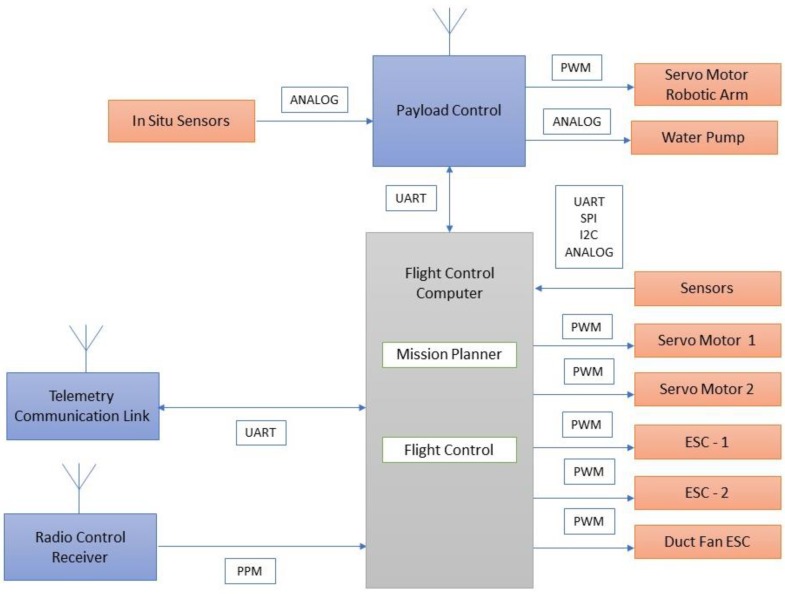
Water sampling mission.

During hover mode of the vehicle (Figure 17), the payload control unit was triggered to actuate the robotic arm, water pump, and water quality sensors to collect water samples and perform in situ water quality analysis.

## 8. Fabrication and Assembly of Amphibious Structure

**Figure 18:**
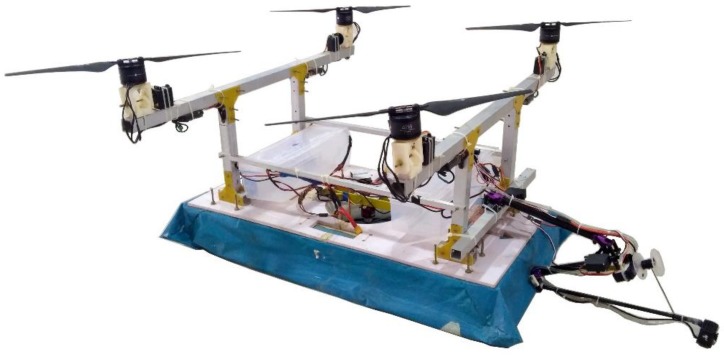
Prototype of amphibious UAV.

**Figure 19:**
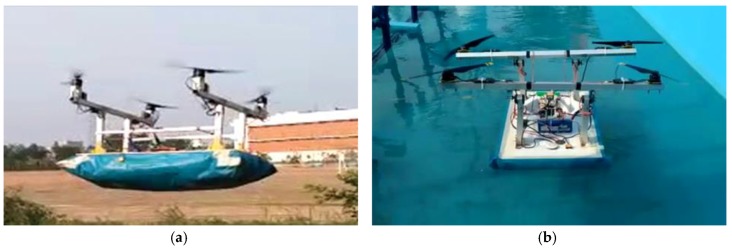
Field testing of amphibious UAV: (**a**) Amphibious UAV (airborne); (**b**) Amphibious UAV (gliding above water).

An amphibious vehicle structure was fabricated (Figure 18) based upon the selected motors, propellers, battery, hull, and skirt materials. On top of the hull, vertical hollow aluminum frames were mounted upon which horizontal frames were fixed. At the four corners of the horizontal frame, 3-D printed knuckle joints were used and a motor-propeller configuration was mounted on it. A servo motor is attached to rotate the motor-propeller configuration. At the center of the hull, a propeller was mounted that produced the necessary pressure to lift the vehicle through inflating the skirt. An open source advanced level controller was utilized to control and navigate the vehicle. The constructed amphibious vehicle was tested in an ambient environment and stable flight was observed (Figure 19a). Preliminary testing of the vehicle was also taken up in a water tank (Figure 19b) and water was collected through actuating the suction pump.

The folded robotic arm was extended through a servo actuator after landing the vehicle on the water surface and a rope mechanism attached with a suction pump was actuated to suck the water at the desired depth of the water channel. After performing the waterborne mission, the robotic arm assembly was retracted to the initial folded configuration for compactness and stability of the vehicle.

## 9. Internet of Things (IoT)-Based Water Quality Measurement

**Figure 20:**
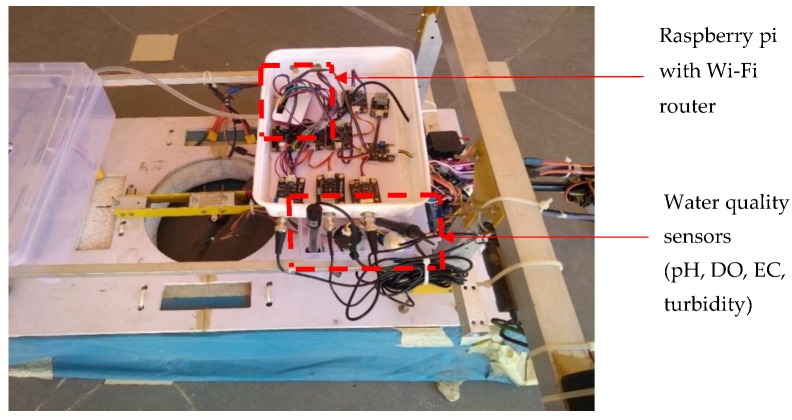
Sensor interface with Raspberry pi.

It is essential to perform water quality inspection at regular intervals at the water reservoirs such as dams, lakes, rivers, and ponds. Collection of water samples in remote water bodies is challenging and time consuming. Traditional methods of collecting water samples using boats is cumbersome and it is very difficult to access remote water locations. In this work, we developed an amphibian vehicle that can measure the water quality using various on-board water quality sensors such as pH, dissolved oxygen (DO), electrical conductivity (EC), temperature, and turbidity, and they were procured. A Raspberry pi zero BCM 2835, 1 GHz ARM 11 core, 512 MB of LPDDR2 SDRAM (Raspberry PI Foundation, Cambridge. UK) was utilized to process the sensor data and sent via a 4G dongle-LTE network at 2300 MHz. The IoT setup shown in Figure 20 was embedded into the designed amphibious UAV and in situ water quality measurements were performed. The sensor data were sampled at a 160 MHz sampling speed using an Arduino pro mini and transmitted to the Raspberry pi UART section. The Raspberry pi was connected with a 4G-LTE dongle, and the UAV was operated with a 2.4 GHz radio frequency to avoid inference. 

The sensor module encompassed pH, dissolved oxygen (DO), electrical conductivity (EC), temperature, and turbidity, which was immersed into the collected water sample during the deployment of the amphibious vehicle in a desired location. Initially, water quality sensors were calibrated with corresponding acidic solutions. The pH sensor was immersed in a KCl solution, the DO sensor tip was stored in NaoH solution, and EC was calibrated with distilled water. These calibrations were performed for each mission before in situ measurement. Once the water quality was measured for a particular sample, water was drained and a new water sample was collected in a container. The sensors were flushed with distilled water for each measurement to obtain accurate results. The measured analog signal was sent to the embedded computer as 16 bit digital data.

**Figure 21:**
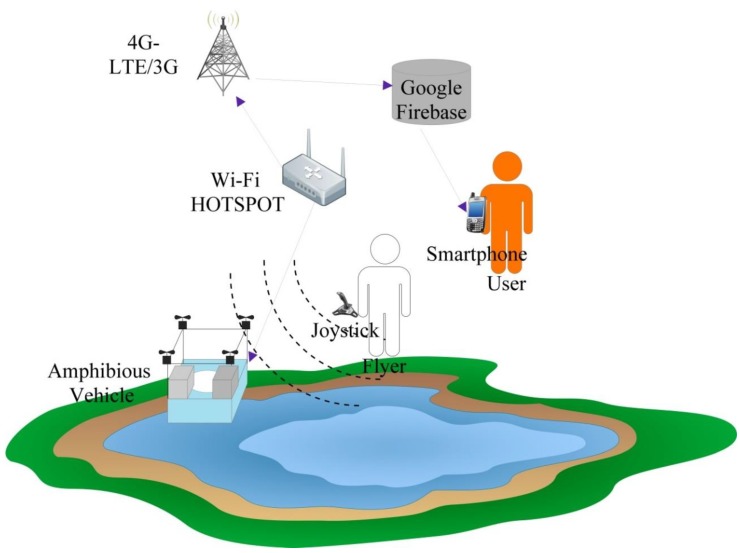
IoT architecture for water quality measurement.

A typical IoT-based network is shown in Figure 21, which demonstrates the working principle of the UAV-based water quality measurement and transmission of data.

**Figure 22:**
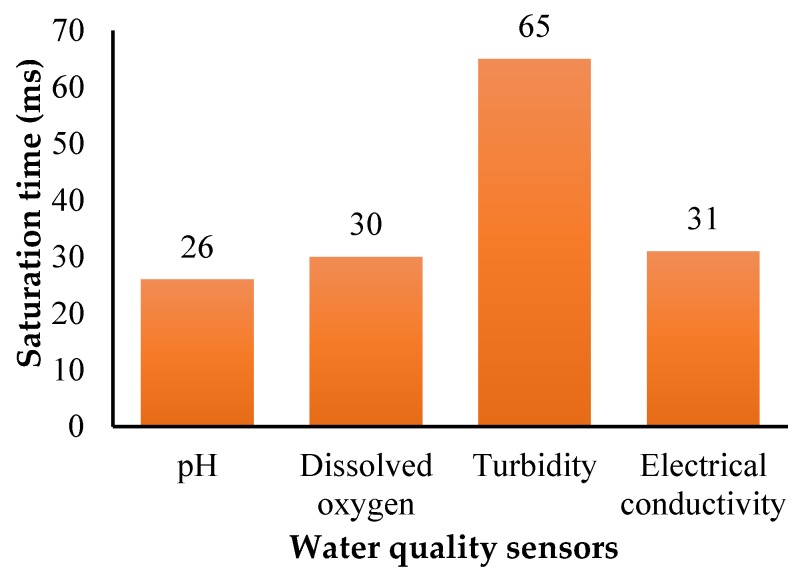
Sensor saturation time.

The ARM-based computer processed the sensor data and provided the useful information for measurable quantities in standard units. The water quality information was transferred to the cloud database for real-time monitoring and post-processing. The link between the cloud data base server and real-time on-board embedded computer were created with the 4G broadband cellular network because it provided better speed in comparison to 2G and 3G. The water quality data in the cloud could be accessed through the smart devices with an Internet service anywhere in the world. Preliminary experiments were conducted to examine the performance characteristics of the sensor network. The saturation time taken for each sensor was obtained and turbidity took more time to arrive at saturation in comparison with other sensors, as shown in Figure 22.

**Figure 23:**
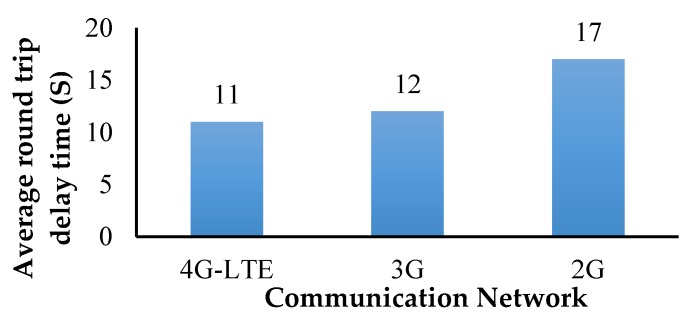
Round trip time.

The average delay (500 set transmissions) in water quality data was monitored with different network conditions (4G, 3G, and 2G). The round-trip delay time seemed to be better for 4G-LTE communication, and it took 11 s on average to reach the destination (Figure 23).

**Figure 24:**
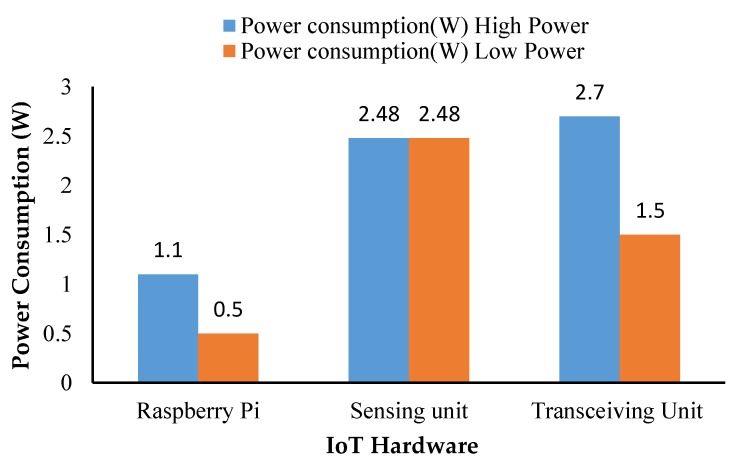
Power consumption of IoT system.

In addition, power consumption of various sensors, and the transmitting and receiving unit was calculated and it is shown in Figure 24.

**Figure 25:**
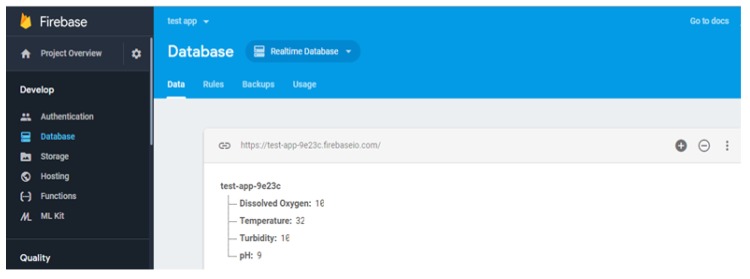
Google fire database.

The transmitted sensor data was collected in the Google firebase cloud as shown in Figure 25 and it could be synchronized with the cloud messaging service and shared across the globe through the firebase cloud services.

**Figure 26:**
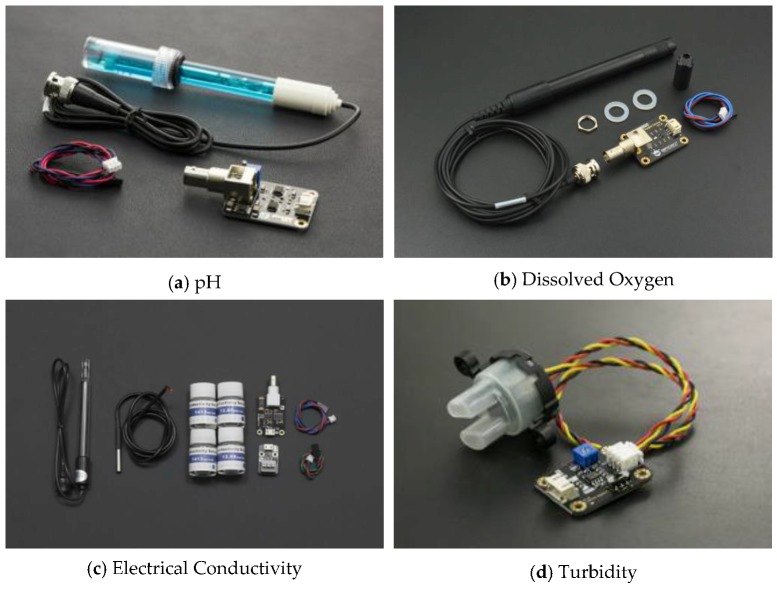
Water quality sensors.

Water quality analysis was performed using the various sensors shown in Figure 26. Water samples were collected at a typical lake near to Ambattur, Chennai, India (13°06′27.9″ N, 80°08′42.0″ E) extending over an area of approximately 1.57 km^2^ and has a total length of 6.06 km. A robotic arm with a water-sucking pump was used to collect the water and it was stored in a container. The complete sensor module was isolated to avoid interference of the signal, and their probes were immersed into the container. In order to demonstrate usefulness of the developed amphibious vehicle, water sampling measurements were carried out at five different water locations across the lake. The real time data was transmitted for a period of time until saturation occurred. The measured data was compared with their upper limit (Table 12) based on the Indian Standard (IS) 10500 water quality standards. It is adopted by the Bureau of Indian Standards approved by the Drinking Water Sectional Committee and also the Food and Agriculture Division Council. In situ measurement showed that all the measured data were almost equal as observed in Table 12. The maximum saturation time of 65 ms in the case of the turbidity sensor was obtained and a minimum of two minutes interval was given between each sensing. The collected water samples were also tested in the lab environment and the test results were in close agreement with the in situ measurement having a 98% accuracy. It is evident from Table 12 that the lake water was of poor quality and it needed water treatment to improve the water quality. 

## 10. Conclusions

An amphibious vehicle was developed for a mission endurance of 25 min while carrying a payload of 7 kg. Design of the vehicle combined the functionalities of a multi-rotor UAV and a hovercraft. Through engineering analysis and simulations, performance of the vehicle was evaluated with reference to deformation, stresses, forward velocity, and stagnation pressure corresponding to expected operational conditions. Appropriate selection of materials for obtaining superior strength characteristics, motors, and propeller to generate sufficient thrust forces and considering a two DOF robotic arm integrated with a water-sucking pump, a prototype was built and tested in an airborne condition (open field) and also in a water body to evaluate the stability and response. The developed amphibious system was able to collect water samples of 500 mL through actuating the suction pump attached at the end-effector of the robotic arm. IoT-based water quality analysis revealed that within 11 ms, the 4G-LTE network transmitted the data to the ground station through firebase cloud services. The developed IoT hardware unit consumed 7.58 W of power and each sensor saturation limit was measured. The turbidity sensor took 65 ms to reach saturation and the pH required 26 ms to attain saturation of the sensed data. Water quality analysis results suggested that, as per IS 10500 water quality standards, the inspected lake water was impure and may not be suitable for drinking purposes.

## Figures and Tables

**Table 1:** Assumptions of the hovercraft parameters.

Serial No.	Empirical Relation	Limit
1	Length to width (*l*/*w*)	2
2	Bag pressure to cushion pressure (*P_b_*/*P_c_*)	1.3
3	Forward thrust to overall weight during hovering (*T_f_*/*W*)	0.2
4	Propeller pitch to diameter (*p*/*d*)	0.6
5	Vertical thrust to maximum take-off weight (*T_v_*/*W*)	2

**Table 2:** Calculation of the hovercraft parameters.

Parameter	Empirical Relation	Values
Maximum take-off weight (*W*)	m×g	269.78 N
Length of the hovercraft (*l*)	2×w	1.00 m
Cushion Area (*A_c_*)	$l\times w-\pi r^{2}$	$0.40\;m^{2}$
Cushion pressure (*P_c_*)	$\frac{W}{A_{c}}$	674.44 N/m^2^
Air escaping velocity (*V_e_*)	$\sqrt{2\frac{P_{c}}{\rho }}$	33.18 m/s
Air escaping area (*A_e_*)	2×(l+w)×h	0.038 m^2^
Air flow rate (*Q_e_*)	$A_{e}\times V_{e}$	1.26 m^3^/s
Power required (*P_e_*)	$\frac{Q_{e}\times \rho \times {V_{e}}^{2}}{2}$	852.32 W

*m*—mass of the hovercraft; *w*—width of the hovercraft; *l*—length of the hovercraft; ρ —air density; *h*—hovering height.

**Table 3:** Mechanical properties of skirt.

Property	Nylon Impregnated with Urethane	Natural Rubber-Coated Nylon	Vinyl-Coated 1000 Denier Polyester
Tensile strength (MPa)	45	35	3.06
Elastic modulus (MPa)	1.48	1.10	20.00
Density (kg/m^3^)	900	1016	1500
Hardness	75	34	87
Flexural strength (MPa)	41	20	26

**Table 4:** Mechanical properties of hull.

Property	Composite Material	Fiberglass	Polyurethane Foam
Tensile strength (MPa)	1200	1950	1900
Compressive strength (MPa)	866	4000	48
Elastic modulus (GPa)	45	72	4
Density (kg/m^3^)	7850	2540	1390
Flexural strength (MPa)	146	110	57

**Table 5:** Multi-rotor structure parameters.

Parameters	Empirical Relation	Values
Required motor speed (*N*)	$\sqrt{\frac{L_{m}\times 10^{10}}{p\times d^{3}\times 0.0283495\times g}}$	4200 rpm
Thrust per motor (*T*)	$p\times d^{3}\times N^{2}\times 10^{-10}\times 0.0283495\times g$	92 N
Lift required for the multicopter (*L_m_*)	2×W	540 N

*L_m_*—multicopter lift required; *p*—propeller pitch; *d*—propeller diameter; *g*—acceleration due to gravity.

**Table 6:** Propulsion system parameters.

Parameters	Empirical Relation	Values
Speed of motor (*N*)	Kv×V	4800 rpm
Operating current (*I*)	$\frac{P}{V}$	50 A
Number of battery cells (*nS*)	$\frac{V}{3.7}$	10
Endurance (*E*)	*mAh* × 0.001 × 60/Σ *I_m_*	25 min

*Kv*—motor rating; *V*—operating voltage; *P*—motor power; *mAh*—milliampere hour; *I_m_*—summation of current consumption.

**Table 7:** Estimation of power and current of on-board electronics.

Sl. No	Component Name	Power Required (W)	Current Consumption (A)
1	Flight controller board	16	0.34
2	ESC	40	0.83
3	Video telemetry	15	0.34
4	Camera	10	0.21
5	On-board processor	50	1.01
Total		131	2.73

**Table 8:** Power consumption (airborne mode).

Sl. No	Components	Power (W)	Total Power Consumption (W)	Total Current Consumption (A)
1	Electronics components	95.30	95.30	2.73
2	Motors	460.80 (per motor)	3686.40 (for eight motors)	76.40 (for eight motors)
Total			3781.70	79.13

**Table 9:** Power consumption (gliding on water).

Sl. No	Components	Power (W)	Total Power Consumption (W)	Total Current Consumption (A)
1	Electronics components	95.30	95.30	2.73
2	Motors	240.00 (per motor)	1920.00 (for four motors)	40.00 (for four motors)
3	Ducted Motor	100.00 (per motor)	100.00 (for one motor)	2.08 (for one motor)
Total			2115.30	44.81

**Table 10:** Weight of each components

Sl. No.	Components	Weight (kg)
1	Multicopter frames (aluminum alloy 6061)	1.40
2	Hull (Polyurethane foam)	0.80
3	Skirt (Nylon impregnated with urethane)	1.70
4	Control system	0.45
5	Multicopter motor	3.36
6	Multicopter propeller	0.31
7	Multicopter electronic speed controller (ESC)	0.88
8	Servo	0.50
9	Electronic duct fan (EDF)	0.40
10	EDF ESC	0.11
11	Li-Po batteries	10.00
12	Miscellaneous	0.40
13	Payload	7.00
Total Weight		27.31

**Table 11:** Drag Estimation.

α Angle of Attack	C_d_ Drag Coefficient	D Drag (N)
**0°**	5.89	38.3
**4°**	5.65	36.8
**8°**	5.50	35.8

**Table 12:** Comparison of water quality with reference to IS 10500 standards.

Serial No.	Sensors	Locations					Maximum Allowable Limit
		L1	L2	L3	L4	L5	
1	pH	8.12	7.98	7.83	8.00	7.01	(acceptable range = 6.5 to 8.5) >7.0 + = alkalinity <7.0 − = acidity
2	Turbidity (NTU)	8.47	8.04	8.01	8.94	8.85	5.0
3	Electric conductivity (ms/cm)	1.31	1.31	1.34	1.37	1.34	0–0.5 mS/cm Good 0.5–1.5 mS/cm Normal >1.5 mS/cm High
4	Dissolved oxygen (mg/L)	8.75	8.75	9.04	8.82	8.61	Above 6 mg/L
